# *In Silico* Elucidation of the Recognition Dynamics of Ubiquitin

**DOI:** 10.1371/journal.pcbi.1002035

**Published:** 2011-04-21

**Authors:** Dong Long, Rafael Brüschweiler

**Affiliations:** Chemical Sciences Laboratory, Department of Chemistry and Biochemistry and National High Magnetic Field Laboratory, Florida State University, Tallahassee, Florida, United States of America; National Cancer Institute, United States of America and Tel Aviv University, Israel

## Abstract

Elucidation of the mechanism of biomacromolecular recognition events has been a topic of intense interest over the past century. The inherent dynamic nature of both protein and ligand molecules along with the continuous reshaping of the energy landscape during the binding process renders it difficult to characterize this process at atomic detail. Here, we investigate the recognition dynamics of ubiquitin via microsecond all-atom molecular dynamics simulation providing both thermodynamic and kinetic information. The high-level of consistency found with respect to experimental NMR data lends support to the accuracy of the *in silico* representation of the conformational substates and their interconversions of free ubiquitin. Using an energy-based reweighting approach, the statistical distribution of conformational states of ubiquitin is monitored as a function of the distance between ubiquitin and its binding partner Hrs-UIM. It is found that extensive and dense sampling of conformational space afforded by the µs MD trajectory is essential for the elucidation of the binding mechanism as is Boltzmann sampling, overcoming inherent limitations of sparsely sampled empirical ensembles. The results reveal a population redistribution mechanism that takes effect when the ligand is at intermediate range of 1–2 nm from ubiquitin. This mechanism, which may be depicted as a superposition of the conformational selection and induced fit mechanisms, also applies to other binding partners of ubiquitin, such as the GGA3 GAT domain.

## Author Summary

Molecular recognition plays a central role in many biological processes, ensuring specific and efficient interaction between binding partners. Various models for describing the mechanisms of molecular recognition have been proposed, but the validation of these models has been traditionally difficult due to the transient and complex nature of the dynamic recognition process. In the present study, we aim at visually characterizing the mutual interplay between human ubiquitin and its ligands via microsecond time scale molecular dynamics simulation, which is validated rigorously against experimental NMR data. Taking advantage of Boltzmann sampling of molecular dynamics snapshots, we statistically reweight the populations of ubiquitin in the presence of its ligand molecule at intermediate distance range (1–2 nm) to examine the population redistribution mechanisms. These results offer new atomistic insights into this vital protein-protein recognition event.

## Introduction

The process of molecular recognition and its fundamental role for protein function have been recognized since the late 19^th^ century. Over time, favored mechanistic models have shifted from the ‘lock-and-key model’ [1] to the ‘induced fit model’ [2] and most recently to ‘conformational selection’ [3]–[5] in order to explain a myriad of binding events including molecular recognition, allosteric regulation, and enzymatic activity. These processes display a variable degree of binding specificity between a protein and one or several small ligands or macromolecules. Recent advances in experimental NMR spectroscopy [6], and computation [7] strongly suggest that conformational dynamics is an essential property of proteins with direct consequences for their function. In the ‘induced fit model’, the ligand first binds loosely to the protein in its inactive form gradually inducing a change to the active form leading to the formation of the final complex. The alternative ‘conformational selection model’ assumes that the unbound protein visits through thermal fluctuations from time-to-time the active forms to which the ligands then binds, resulting in the final dynamically restricted protein-ligand complex. Most recently, a generalization of the conformational selection model that encompasses both the selection and adjustment features has been proposed [8]. In addition, based on coarse-grained simulations [9], [10] mechanisms intermediate between conformational selection and induced fit were suggested. However, because the details of the binding process are difficult to capture directly by measurements, these models have not yet been validated by experiment. NMR studies of free protein states often identify signatures of the bound state pre-existing even in the absence of the ligand [11]–[14]. While such a behavior is consistent with the conformational selection model, it may not necessarily rule out an induced fit process as an alternative mechanism [15].

In a recent pioneering study, Lange et al. [16] put the intrinsic flexibility of ubiquitin, inferred from NMR residual dipolar couplings (RDCs), in relationship to the structural diversity of a set of X-ray crystal structures of ubiquitin complexes with other proteins. An empirical ensemble consisting of 116 structures, termed EROS, generated from RDCs and other NMR data of free ubiquitin was found to cover well the structural heterogeneity of ubiquitin in the various complex forms, which supported the notion of conformational selection for the binding process of ubiquitin. Re-interpretation of the same data by Wlodarski and Zagrovic [17] suggested that during the binding event the protein conformation that is structurally most similar to the bound conformation becomes predominant via the conformational selection model. This step is followed by optimization of the binding interface via a ‘residual induced fit’ mechanism after the conformational selection step. Both studies [16], [17] relied on the interpretation of the free state of ubiquitin by a relatively small conformational ensemble, but did not take into account motional time scale effects reflecting interconversion rates between conformers nor the alteration of the protein energy landscape itself by the presence of the binding partners.

A detailed thermodynamic and kinetic picture in terms of populations of individual protein conformations and the time scales of their interconversions is essential for understanding the recognition dynamics in a biological context and also to discriminate between induced fit, conformational selection, or other mechanisms, but unfortunately such information is not provided by empirical ensembles [16], [18]–[22]. The goal of the present work is to obtain a detailed mechanistic view of the binding process of ubiquitin by interpreting the experimental data based on substantially larger, time-resolved ensembles that obey Boltzmann statistics. Recent advances in computer hardware and molecular mechanics force fields has permitted the *in silico* investigation of protein dynamics with unprecedented accuracy on increasingly long time scales [7], offering a powerful tool for studying mechanisms of molecular recognition [23]–[26]. In this study, we perform microsecond time scale all-atom molecular dynamics (MD) simulations of ubiquitin and its binding partners using the latest generation of molecular mechanics force field [27], which provides new qualitative and quantitative insights into the recognition dynamics of this model system.

To examine the effect of ligand molecules, we focus here on the ubiquitin interacting motif (UIM), which is a conserved short α-helical motif for ubiquitin recognition found in many proteins involved in ubiquitin association [28],[29]. Recent NMR relaxation data reveal the capability of a UIM domain to perturb the plasticity of the ubiquitin molecule on multiple time scales [30]. In the present study, we use the ubiquitin:Hrs-UIM complex [31] to examine at atomic detail the energy landscape and dynamics of ubiquitin in response to the approach of the Hrs-UIM ligand.

## Results

### Validation of the microsecond MD trajectory against NMR parameters

To achieve comprehensive and accurate sampling of the conformational space of free ubiquitin, a 1 µs MD simulation was performed in explicit solvent at 300 K. Backbone residual dipolar couplings measured in 22 alignment media and chemical shifts of ubiquitin, which encode atomic-detail information of protein structure and dynamics, are back-calculated from the MD trajectories using methods discussed previously [32], [33] and compared to the experimental values [34], [35] for validation (Figures S1 & S2). The observed level of agreement with this extensive body of experimental data attests to the good accuracy of the MD ensemble permitting the extraction of quantitative information about conformational substates and their thermal fluctuations from the trajectory as explained below.

### Sampling of conformational space of free ubiquitin

**Figure 1:**
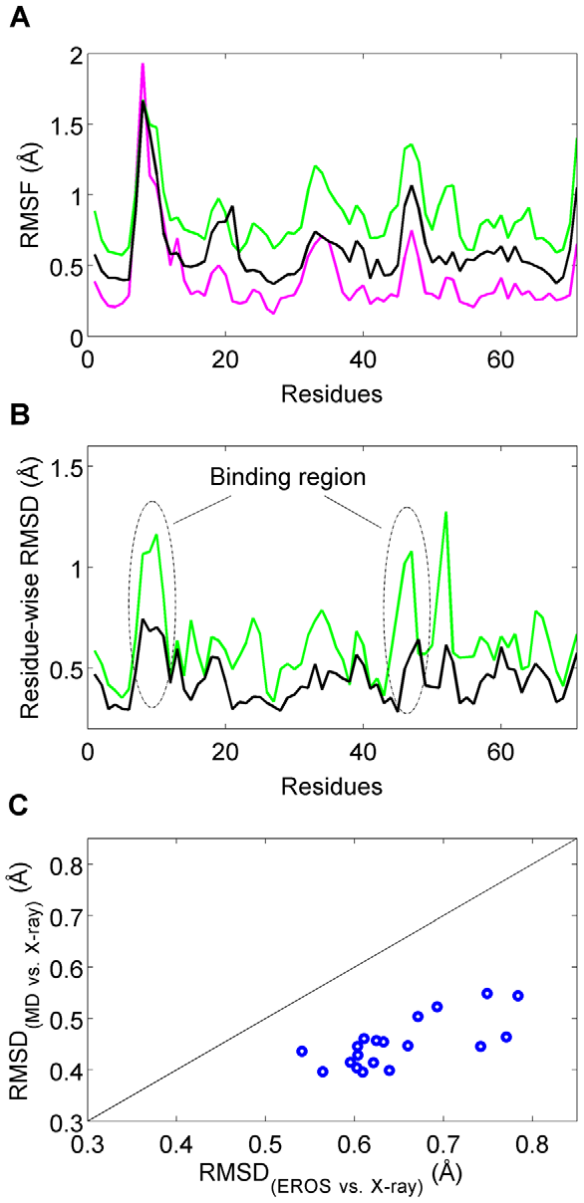
Structural analysis of X-ray crystal structures, EROS ensemble, and MD ensemble. (A) Cα root mean square fluctuation (RMSF) of MD ensemble (black), EROS ensemble (green), and 19 ubiquitin X-ray crystal structures (magenta). (B) Residuewise root mean square deviation (RMSD) values (Eq. (1)) for the pair-wise structural comparisons of X-ray crystal structures and their closest EROS conformers (green solid line) and X-ray crystal structures and their closest MD conformers (1 million structures) (black solid line). Green peaks that belong to protein binding regions are circled with dash-dot lines. (C) The Cα RMSD calculated for each X-ray crystal structure with the closest EROS conformer (x-axis) and the closest MD conformer (y-axis). The RMSD values of the MD conformers are substantially smaller than for the EROS conformers. Definitions of RMSF and RMSD parameters are given in Text S1.

Backbone Cα fluctuations, expressed as root mean square fluctuations (RMSF), of free ubiquitin during the course of the MD simulation reveals similar trends as those seen for the EROS ensemble and the X-ray crystal structures of the ubiquitin complexes (Figure 1). Although the MD-fluctuations are on average considerably smaller than the ones of the EROS ensemble, they well capture the structural variability among the crystal structures.

**Figure 2:**
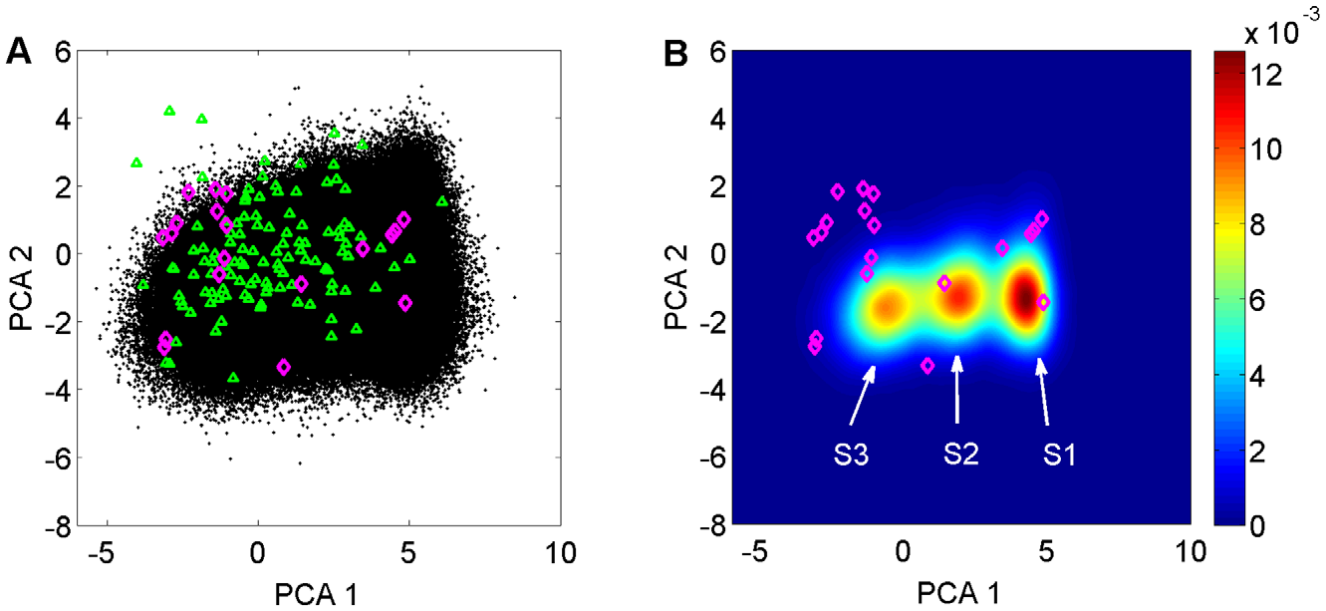
Projection of the structural ensembles on 2D PCA space. (A) Comparison of the X-ray structures (magenta diamonds), EROS ensemble (green triangles) and MD ensemble (black dots) in 2D space spanned by the 2 largest principal components. PCA was performed using the 19 X-ray crystal bound forms only. (B) Population distribution of MD ensemble on two dimensions, which can be grouped into the three substates S1, S2, and S3.

For a better comparison of the different structural ensembles, we projected 19 representative X-ray crystal structures, the EROS ensemble, and the full MD ensemble on the two largest principal components determined from the X-ray crystal structural ensemble. Clearly, the MD ensemble of the free form of ubiquitin samples all the bound crystal structures (Figure 2A) with some of the crystal conformations located in regions that are at the boundary of the conformational space sampled by the MD trajectory and hence they are relatively lowly populated (Figure 2B). The 10^6^ snapshots of the MD ensemble generated during the 1 µs trajectory densely cover the relevant conformational space, which contrasts the sparse coverage provided by the 116-member EROS ensemble (Figure 2A).

### Structural comparison between free ubiquitin and all ubiquitin crystal complexes

A recent pair-wise structural comparison between X-ray crystal structures and the corresponding closest EROS conformers [17] showed that the structural changes in regions surrounding the binding site are significantly more pronounced than for the rest of the protein. With the extended MD trajectory available, this result prompted us to examine whether it is caused by the incomplete representation of free ubiquitin by only 116 EROS structures. Therefore, we conducted pair-wise structural comparisons for both X-ray crystal structures *vs.* the closest EROS conformers and X-ray crystal structures *vs.* the closest MD conformers. A systematic drop of the Cα RMSD is observed for all 19 X-ray structures when a dense representation of the MD ensemble with all 10^6^ snapshots is used (Figure 1C). Moreover, the above-average deviations in the binding regions, which are observed in the comparison of X-ray crystal structures *vs.* closest EROS conformers, disappear when using the MD ensemble (Figure 1B). Sparser sampling of the MD ensemble leads to a behavior that resembles the one of the EROS ensemble (Figure S12). Thus, the full MD simulation provides a qualitatively new picture of the relationship between the free state of ubiquitin and its conformations in the various complexes.

### The principal mode dynamics of free ubiquitin

In the PCA subspace spanned by the two largest modes, the conformations of free ubiquitin can be grouped into three major substates, which are primarily separated along the first principal component (Figures 2B & S5). The internal dynamics along the largest principal mode corresponds to pincer-like motion (Figures S7 & S8) involving regions of the loop β1–β2, loop α1–β3, and the C-terminal of helix α1, consistent with the results by Lange et al. [16].

**Figure 3:**
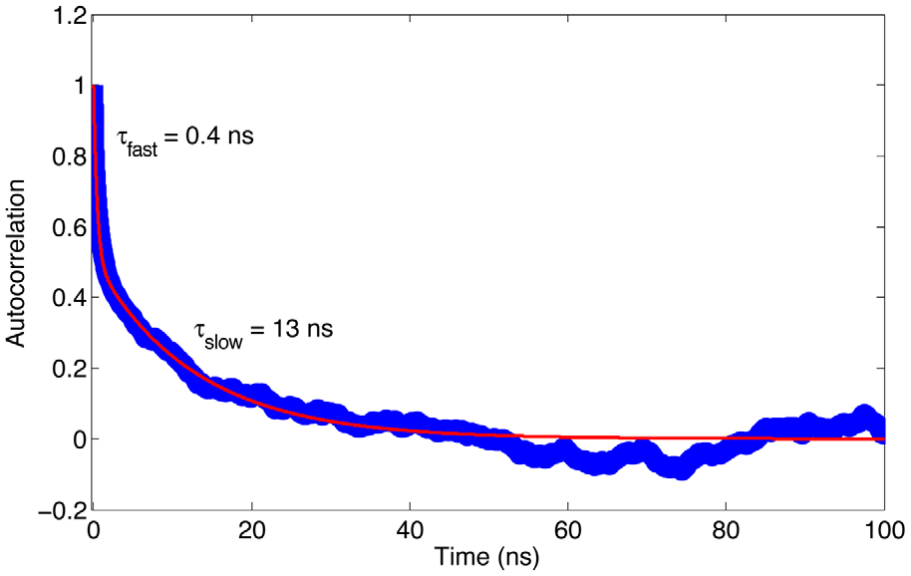
Autocorrelation function of the principal mode motion. The autocorrelation function *C*(*t*) of the internal dynamics along the first principal component (Eq. S5; blue line) is fitted with a two-exponential function (Eq. S6; red line). The parameters extracted by curve fitting are: *a* = 0.49; *τ_fast_* = 0.4 ns; *τ_slow_* = 12.9 ns.

The autocorrelation function *C*(*t*) of protein motion along the largest mode reveals the motional time scales involved (Figure 3). Its accurate characterization requires an expansion with at least four exponentials with effective correlation times ranging from picoseconds to tens of nanoseconds (Figure S6), suggesting that the energy landscape has a rugged nature with multiple energy barriers of various heights. The two dominant exponentials have effective correlation times of 0.4 ns and 13 ns, respectively. Appropriate sampling of all three substates in a single continuous trajectory requires a simulation length that exceeds the correlation time by typically two orders of magnitude. Indeed, the average agreement between calculated and experimental backbone ^15^N-^1^H RDCs and chemical shifts, expressed by the average Q value and chemical shift RMSDs, respectively, steadily improves as the trajectory approaches the µs-range (Figure S3). While all three sub-states, S1, S2, S3, have a similar population (Figures 2 & S5), S3 is closer to the majority of crystal-bound conformations than S1 and S2 and therefore is designated to play a unique role in the binding event as evidenced by the following analysis.

### Population shift in the presence of Hrs-UIM

**Figure 4:**
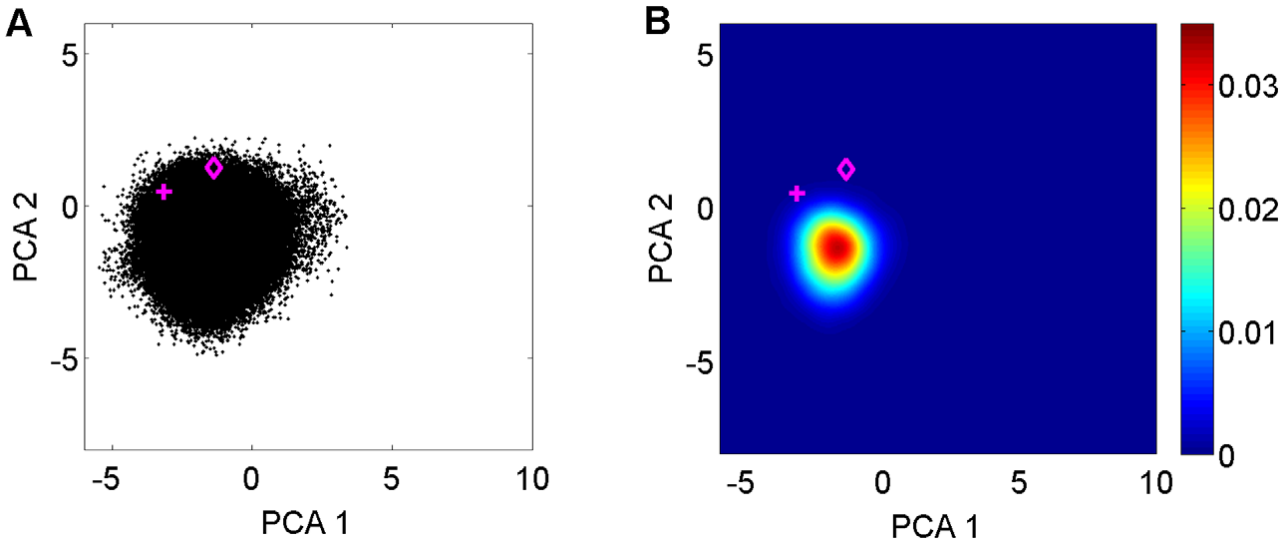
Conformational space accessible to ubiquitin in complex with Hrs-UIM. (A) 2D projection of complex MD ensemble (black dots). The corresponding crystal conformations are indicated by the magenta diamond for chain A (PDB code: 2D3G) and the magenta ‘+’ symbol for chain B of the same PDB entry. (B) Pseudo color presentation of population distribution of the ubiquitin bound form.

In order to obtain the macrostate ensemble of the stable ubiquitin:Hrs-UIM complex, a 300 ns explicit-solvent MD simulation of this complex was performed starting from the X-ray crystal structure [31]. It shows that the bound state adopts in principal component space a distribution that is similar to the one of substate S3 of free ubiquitin (Figures 4 & S9). This result suggests that the presence of Hrs-UIM specifically selects a sub-ensemble of ubiquitin conformations that have a favorable shape and interaction properties during molecular recognition.

**Figure 5:**
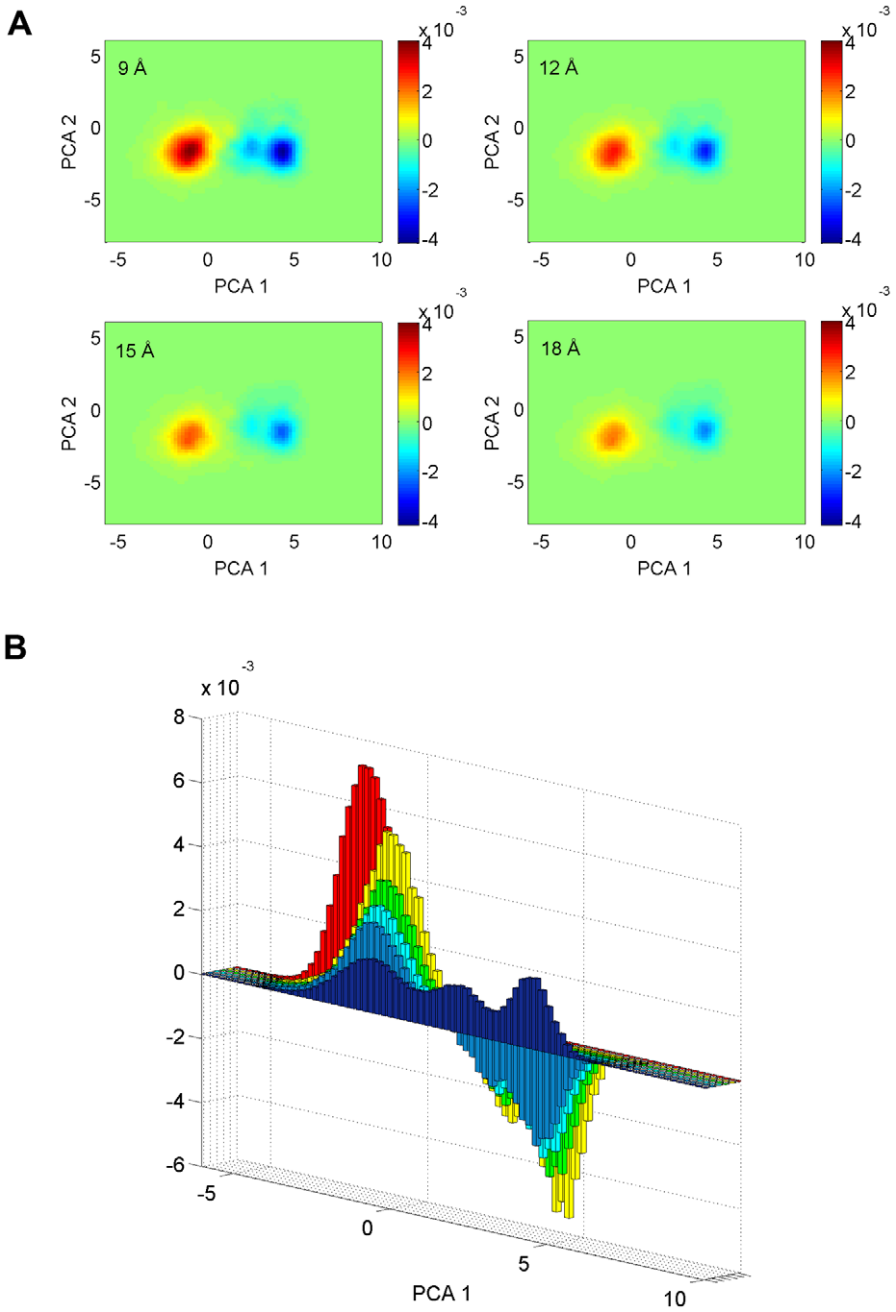
Population redistribution of substates of ubiquitin upon the approach of Hrs-UIM. (A) The two-dimensional difference maps of the population densities were calculated in the presence of Hrs-UIM in the range from 9 to 18 Å away from its position in the crystal complex (PDB code 2D3G, chain P). (B) One-dimensional projection of the population difference maps along the largest principal component. Hrs-UIM was introduced at distances of 9 Å (yellow), 12 Å (green), 15 Å (cyan), and 18 Å (light blue). The ubiquitin populations in the free (dark blue) and bound (red) forms are included for comparison. For clarity, the populations of the free and bound states are scaled by 1/19 and 1/13 in the plot. It can be seen that the substate S3 becomes increasingly populated as Hrs-UIM approaches the binding site.

**Figure 6:**
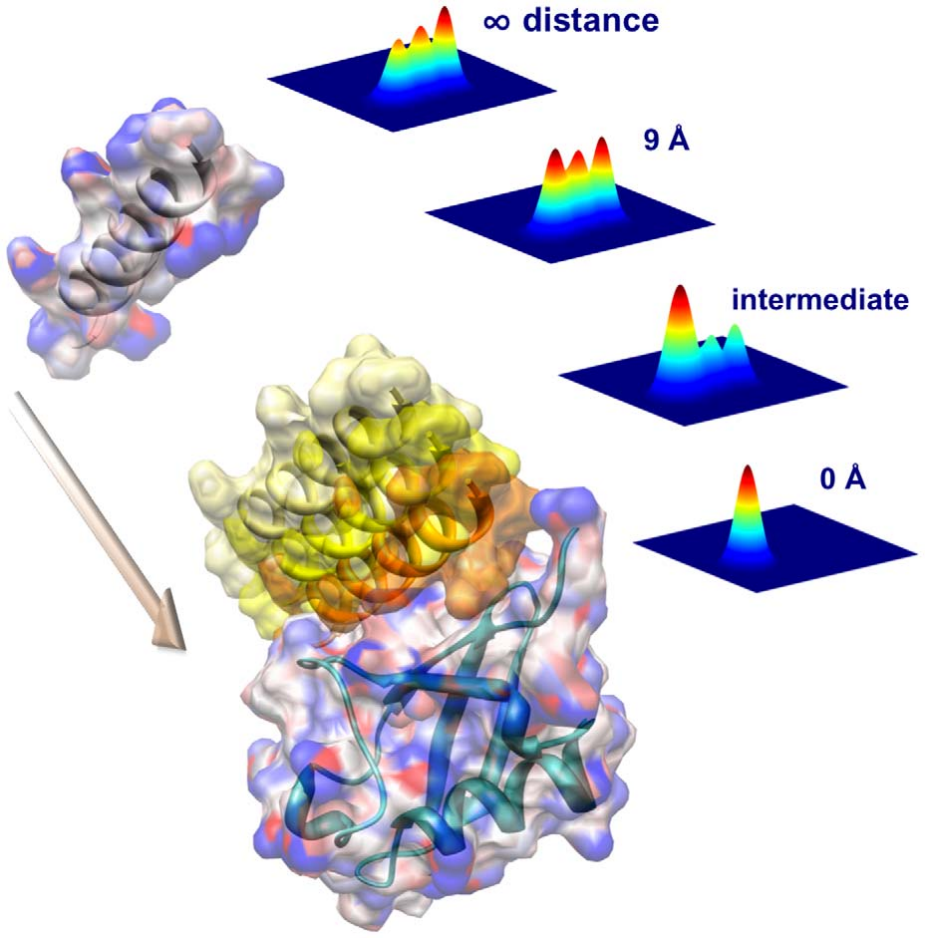
Schematic illustration of the population redistribution upon the approach of Hrs-UIM to the binding interface. The population distribution at an intermediate distance (between 0 and 9 Å) is represented by the linear combination of the population distributions at 0 and 9 Å with weights of 30% and 70%, respectively. The four population density plots have the same axes as Figure 2B. The reshaping of the energy landscape of ubiquitin occurs in a continuous way, starting at nanometer distance range between the binding partners.

The large pool of ubiquitin structures from the 1 µs MD simulation of free ubiquitin that encompasses all the bound conformations allows us to systematically analyze the response of ubiquitin to the presence of Hrs-UIM. Application of Boltzmann reweighting of individual conformers [36], by recalculating their potential energies as a function of the distance of Hrs-UIM from the binding site, enables us to study population shift of ubiquitin at unprecedented detail (Figures 5 & 6). While the perturbation of ubiquitin populations by Hrs-UIM at long distances is subtle, the population difference map unequivocally shows that the favored, i.e. dominant, conformations are gradually shifted toward the bound state. In particular, substate S3 becomes increasingly populated as Hrs-UIM approaches the binding site. Remarkably, ubiquitin already experiences a significant bias toward the macrostate ensemble of the final bound form even when Hrs-UIM is at nanometer distance (9–18 Å) from the final bound position. This behavior is robust with respect to moderate changes of the angle of approach of the Hrs-UIM ligand (Text S1 & Figure S11). Analysis of individual contributions to the total energy shows that at large distance range (18 Å) the population shift is dominated by electrostatic interactions while van der Waals interactions come into play at shorter distances only.

## Discussion

Conformational selection and induced fit are two models at opposite ends of a spectrum of mechanisms hypothesized for protein-ligand binding. From a statistical mechanics perspective, the Boltzmann population of any conformer is always non-zero, irrespective of its potential energy. Hence, any conformation, including any bound conformation, has a finite probability to pre-exist in the free state. From a biological perspective, however, the limited sampling of conformers during the lifetime of a biomolecule imposes a natural energy threshold for the biological relevance of high-energy conformers. In the biochemical literature [16], [23], [37], conformational selection is typically invoked when the populations of pre-existing bound conformations are sufficiently large to be detectable in experiments or simulations; otherwise, induced fit is the preferred scenario. The latter mechanism requires a certain degree of plasticity of the protein, but does not take into account thermal fluctuations, i.e. protein dynamics, involving transient populations of multiple protein conformations. The classical conformational selection mechanism, on the other hand, addresses thermal fluctuations, while the alteration of the protein energy surface due to the spatial vicinity of the ligand during the binding process is not emphasized.

Our results of ubiquitin-UIM binding point to an intricate interplay of the two mechanisms for this model system. The induced fit mechanism provides an appropriate description of the binding process if for a given protein-ligand distance only the average structure of ubiquitin is considered. In its free state, the average structure of ubiquitin corresponds to the center of S2, while a gradual shift toward S3 is induced as Hrs-UIM approaches the binding site. By contrast, from an ensemble perspective, where all available substates are considered for a given protein-ligand distance, the conformational selection mechanism is more appropriate. Hence, distinction between the two models depends on the resolution at which ubiquitin is viewed: induced fit prevails at the level of the time- or ensemble-averaged structure, while conformational selection accompanied by energy landscape adjustment is a better model for an ensemble description.

Our study is based on microsecond time scale all-atom simulations that are validated against experimental NMR data. This approach differs in philosophy from previous work that built a structural ensemble (EROS) directly from NMR data [16]. The MD approach used here generates a conformational ensemble that obeys Boltzmann sampling and its time-dependence reflects genuine dynamics. In this way, both spatial and temporal atomic-detail information is gained about the recognition dynamics. A recent analysis of the EROS ensemble and X-ray structures determined statistically pronounced structural differences in the binding regions between the free and bound forms of ubiquitin, and a residual induced fit mechanism was proposed to explain this effect [17]. By contrast, the full 10^6^-member MD ensemble used here proves to be sufficiently dense to approach each crystal structure within 0.55 Å RMSD (0.45 Å average) (Figure 1C), with the binding regions behaving in essence indistinguishably from the rest of the protein. Only when the MD ensemble is thinned out 1000-fold or more, a behavior similar to one of the EROS ensemble emerges (Figure S12). Therefore, although the conformational space sampled by the EROS and the MD ensemble does significantly overlap, which is consistent with a recent study using the Amber ff99SB force field [38], the small size of the EROS ensemble leads to a low-resolution depiction of conformational space occupied by free ubiquitin and thereby misses important aspects of the structural variability surrounding the binding regions. A better statistical representation of these regions is provided by the significantly larger µs MD ensemble. Based on the MD ensemble results, a residual induced fit mechanism [17] is not required to explain the structural deviations of the non-tail backbone region.

In addition, the MD results highlight the relevance of kinetic properties (time scales of the conformational interconversions), in addition to equilibrium populations, for ligand binding. Kinetics not only determines how frequently individual states are formed, but also reflects how efficiently the populations can shift toward the bound state in the presence of the ligand binding. In 2D PCA subspace of free ubiquitin, three major conformational substates are identified that undergo a pincer-like motion on the picosecond to sub-microsecond time scales. This reflects some degree of ruggedness of the underlying energy surface with the energy barriers being relatively low. These properties provide the protein a dynamic plasticity permitting rapid structural adaption to medium- and short-range interactions with the ligands and allowing the protein to bind to a host of different binding partners.

Interestingly, although the maximum population density of substate S3 is slightly smaller than for the other substates in the free form of ubiquitin, substate S3 is the closest to the majority of crystal complexes (12 out of 19). This includes the ubiquitin:Hrs-UIM complex examined in this study whose static crystal structure resides in close vicinity to state S3. Moreover, a 300 ns explicit MD simulation of the complex shows details of the dynamic interplay between ubiquitin and Hrs-UIM. Bound ubiquitin has a significant overlap with substate S3, but almost no overlap with substates S1 and S2 (Figure 5B). There is a small offset between the distribution of substate S3 and the final bound form (Figure 5B), which in the framework of an extended conformational selection model [8] can be understood as the result of the reshaping of the energy funnel upon protein-ligand interaction. This result underlines the importance of substate S3 of free ubiquitin for the recognition of Hrs-UIM. However, elucidation of the detailed mechanism of the binding process with Hrs-UIM requires that the response of ubiquitin to the change of its energy surface is studied during the actual docking process.

For this purpose, an energy-based reweighting method is used to map the population density of ubiquitin as a function of the distance of Hrs-UIM from the binding site. A steady increase of the population of S3 is accompanied by a decrease of the populations of both S1 and S2. Therefore, conformational selection by Hrs-UIM at the macrostate level corresponds to the continuous reshaping of the protein energy landscape, gradually favoring the ensemble of the final bound form (Figure 6). This picture may not be adequately described by either the traditional conformational selection model or the induced fit model, both of which, however, cover important aspects of this binding process. A concurrent superposition of the two mechanisms, rather than sequential events [17], is required for a satisfactory explanation, which is noteworthy considering the basic nature of the protein recognition process studied here. This picture is consistent with the framework of extended conformational selection proposed recently [8] and at the same time it provides a fully atomistic view of this key event for ubiquitin.

Dynamic population changes as a function of the distance of an approaching ligand have been proposed in the literature based on simplified models to illustrate the binding mechanism [8], [39]. Unfortunately, such transient dynamics processes are hard to capture and hence confirm by experiment alone. The energy-based reweighting method employed here represents an efficient tool to investigate the gradual change of the energy landscape and provides, to the best of our knowledge, the first quantitative atomic-detail picture of protein population redistribution over a range of distances. It should be emphasized that this approach relies on the fact that the underlying MD ensemble obeys Boltzmann statistics, whereas empirical ensembles that do not obey Boltzmann statistics are not amenable to such a reweighting strategy.

The binding mechanism of Hrs-UIM to ubiquitin identified is not unique. In fact, the GGA3 GAT domain [40], showing a similar binding mode as Hrs-UIM when binding to ubiquitin, also shifts populations toward substate S3 upon approaching the binding interface (Figure S10). Some of the other binding partners of ubiquitin appear to behave in a more complex way. For example, the catalytic domain of USP14 [41] adopts in its bound state a form that sterically hinders the approach of ubiquitin to the binding site. Characterization of the binding mechanism of this type of systems requires a model that simultaneously accommodates structural and dynamics changes of both binding partners [42]. In addition, without prior knowledge of the optimal pathway for ubiquitin to enter the binding pockets, diffusive rotational motion of both proteins needs to be included in the treatment for the full understanding of the binding mechanism. While computationally more expensive, a generalization of the reweighting approach used here to two interacting ensembles that probe multiple orientations seems feasible representing a promising route toward this goal as a complementary approach to brute-force MD simulations.

## Methods

### Molecular dynamics simulation

A 1 µs simulation of the free form ubiquitin was performed at 300 K with AMBER99SBnmr1 force field [27] and TIP3P water model [43] using the GROMACS software package version 4.0.7 [44]. The crystal structure of ubiquitin (PDB code 1UBQ) [45] was employed as the initial conformation. Non-bonded interactions were cut off at 8 Å and the long-range electrostatic interactions were treated using the particle-mesh Ewald summation method [46]. All bonds involving hydrogen atoms were constrained using the LINCS algorithm [47], and an integration time step of 2 fs was used. Prior to the production run at 300 K, the system was relaxed by energy minimization using the steepest descent algorithm, followed by position restrained simulation under NVT conditions for 100 ps and under NPT conditions for another 100 ps. For the 1 µs production run, snapshots were stored every 1 ps, which yields an ensemble with 1 million snapshots.

For the simulation of the ubiquitin-UIM complex in TIP3P water, the starting conformation was built based on the crystal structure of ubiquitin and Hrs-UIM (PDB code: 2D3G, chains A and P, respectively) [31]. Since residues 73–76 of ubiquitin in 2D3G are missing, the ubiquitin conformation of 1UBQ was used for this simulation by superimposing the backbone of residues 2–71 to chain A of the structure 2D3G. The protocol for setting up the simulation was fully analogous to that of free ubiquitin described above.

### Structural analysis

The present structural analysis focused on the backbone core region of ubiquitin (residues 1–71), i.e. without the C-terminal tail residues 72–76. 19 X-ray crystal structures of bound ubiquitin [17] were used for analysis, which were selected from the original 46 crystal structures of [16] and identified to be most representative of all the binding interfaces of ubiquitin complexes. For each X-ray crystal structure the closest conformer in the EROS and MD ensembles was identified based on the overall backbone Cα RMSD. The residue-wise Cα root mean square deviation was then calculated (see also Text S1)
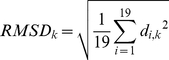
(1)in which *d_i,k_* is the distance between the Cα atom of the *k*th residue of the *i*th X-ray structure and that of the corresponding closest conformer in the EROS and MD ensembles, respectively.

To analyze the structural ensembles in a low dimensional space, all ubiquitin structures (X-ray, EROS and MD) were aligned with respect to 1UBQ. Principal component analysis in Cartesian coordinates was carried out based on the Cα atomic positions of the 19 X-ray structures to highlight the dominant backbone structural changes in the various bound forms (Figure S4). The X-ray structures, EROS, and MD ensembles were subsequently projected onto the first two principal components, which correspond to the modes with the highest variance among all modes. Two-dimensional population maps were constructed with a grid resolution of 0.4 Å and spline interpolation. The total populations were normalized to 1.

### Reweighting of the populations

To examine the gradual shift of the energy landscape in the presence of Hrs-UIM, the ligand in its X-ray structure conformation was translated from its position in the complex for a series of distances (9–18 Å) in a direction orthogonal to the binding interface (Figure 6). For each distance, the population map of ubiquitin conformations was obtained from the original 1 µs trajectory by a reweighting [36] approach based on the potential energy. For each snapshot *j*,
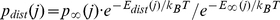
(2)in which, *p_dist_(j)* and *E_dist_(j)* are the relative weight and energy of the system, respectively, in the presence of a ligand molecule at a given distance from the bound position; *p_∞_(j)* and *E_∞_(j)* are the relative weight and energy of the system, respectively, with the ligand at a distance of 1000 Å away from the bound position (representing the limit toward an infinitely large distance). *p_∞_(j)* of all *n* snapshots have equal weights *p_∞_(j)* = 1/*n*. The energy of the combined ubiquitin-ligand complex was calculated for each snapshot after introducing the ligand molecule at a given distance using the implicit generalized Born solvation model [48] as implemented in the Amber 9 software package [49]. The reweighted two-dimensional population maps were constructed with a grid resolution of 0.6 Å and spline interpolation. The total populations were normalized to 1. The 2D difference maps were subsequently calculated with respect to the free ubiquitin.

## Supporting Information

Figure S1 Experimental (RDC_exp_) *vs.* back-calculated (RDC_calc_) backbone NH RDCs of free ubiquitin in 22 distinct alignment media.
(TIF)

Figure S2 Predicted vs. experimental chemical shifts of Cα, Cβ, and C′ nuclei of free ubiquitin.
(TIF)

Figure S3 Dependence of NH RDC Q_av_ values (left panel) and chemical shift RMSDs (right panel) as a function of time window size from 0.1 ns to 1 µs for free ubiquitin.
(TIF)

Figure S4 PCA eigenvalues, expressed as percentages (i.e. the sum of all eigenvalues is 100%) of the ten largest principal components determined from the 19 X-ray crystal structures.
(TIF)

Figure S5 One-dimensional (A–C) and two dimensional (D–F) PCA projections of the 1 µs MD ensemble along the largest principal mode.
(TIF)

Figure S6 Fitting of the autocorrelation function C(t) (blue open circles) with Eqs. S6 (A), S7 (B), and S8 (C), respectively (red lines). To highlight the quality of fits at short time scales, only the time window <10 ns is displayed. The extracted parameters are (A) Eq. S6: *a* = 0.49; *τ_fast_* = 0.4 ns; *τ_slow_* = 13 ns. (B) Eq. S7: *a*_1_ = 0.07; *a*_2_ = 0.09; *a*_3_ = 0.34; *a*_4_ = 0.5; *τ*_1_ = 0.004 ns; *τ*_2_ = 0.08 ns; *τ*_3_ = 0.7 ns; *τ*_4_ = 13 ns. (C) Eq. S8: *a*_1_ = 0.17; *a*_2_ = 0.33; *a*_3_ = 0.5; *τ*_1_ = 0.04 ns; *τ*_2_ = 0.7 ns; *τ*_3_ = 13 ns; *β* = 0.54.
(TIF)

Figure S7 Relative amplitude of Cα positional changes in X-ray structures in the 1^st^ principal component.
(TIF)

Figure S8 Superposition of ribbon representations of the average structures of substates S1 (blue), S2 (yellow), and S3 (red). The conformers within 0.3 Å of the individual maxima of the three substates depicted in Figure S5A were clustered and used for the determination of average structures.
(TIF)

Figure S9 Population distribution of ubiquitin bound to Hrs-UIM sampled at a temperature of 300 K (left panel) and 330 K (right panel). The corresponding crystal bound ubiquitin conformations are indicated by the magenta diamond (for chain A, PDB code: 2D3G) and the magenta ‘+’ symbol (for chain B, PDB code: 2D3G).
(TIF)

Figure S10 Change of ubiquitin populations upon the approach of human GGA3 GAT domain. Difference maps of populations were calculated at 4 distances from 10–18 Å. The crystal conformation (PDB code: 1YD8, chain U) is indicated by the magenta diamond.
(TIF)

Figure S11 Population shift of ubiquitin upon the approach of Hrs-UIM from different directions. The ligand was placed in three different directions with respect to ubiquitin (panel A), as colored in red, cyan, and green. The corresponding population difference maps are shown in panels B, C, and D, respectively.
(TIF)

Figure S12 Residue-wise RMSD between X-ray structures with EROS ensemble (black dashed line) and MD ensembles represented by 1 million (blue solid line), 1000 (light blue), and 100 (green) structures. The three MD ensembles were generated by selecting conformations every 1 ps, 1 ns, and 10 ns, respectively. The binding regions are indicated by magenta dash-dot lines.
(TIF)

Text S1 Supplementary methods and supplementary results.
(DOC)
