# Supplementary figures and images for: In Silico Elucidation of the Recognition Dynamics of Ubiquitin
Source: PLoS Comput Biol. 2011 Apr 21;7(4):e1002035. doi: 10.1371/journal.pcbi.1002035 (PMC3080845; doi:10.1371/journal.pcbi.1002035)

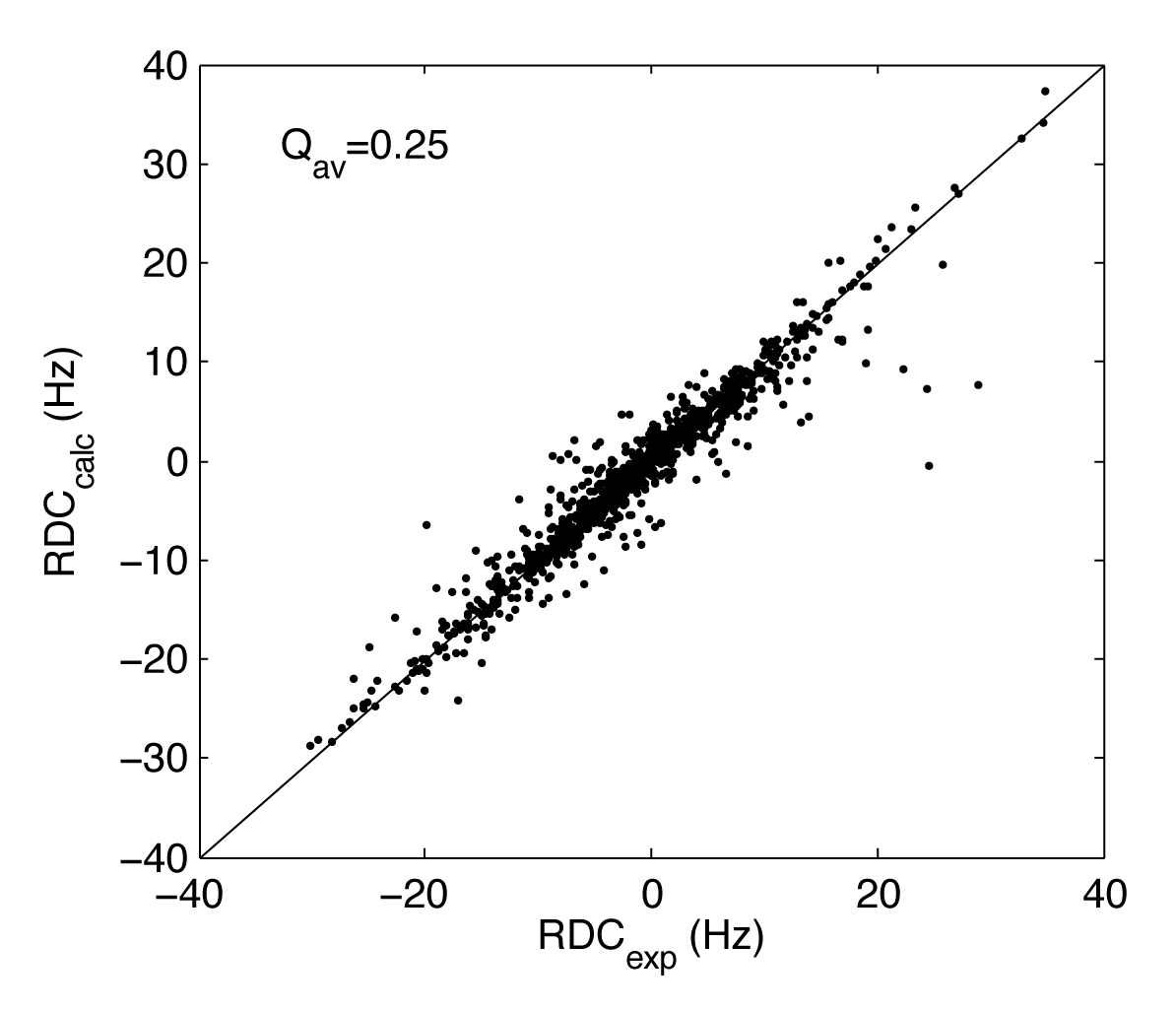

Supplement: Figure S1 — Experimental (RDCexp) vs. back-calculated (RDCcalc) backbone NH RDCs of free ubiquitin in 22 distinct alignment media. (TIF) [file pcbi.1002035.s001.tif]

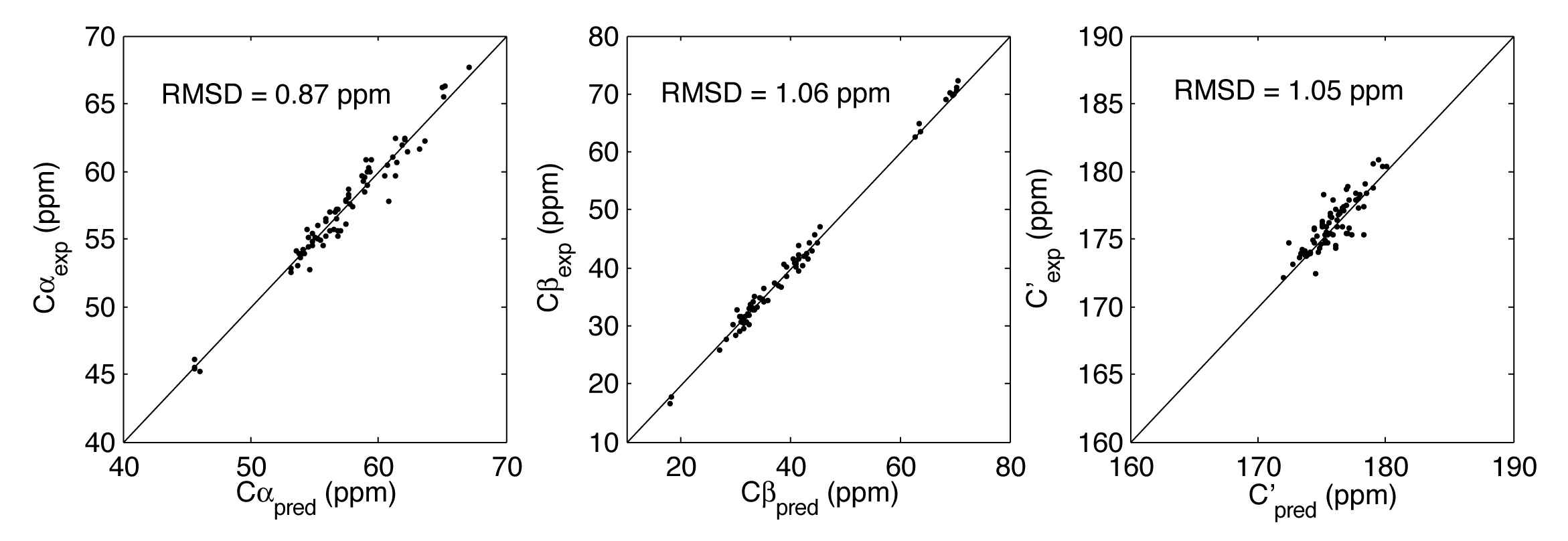

Supplement: Figure S2 — Predicted vs. experimental chemical shifts of Cα, Cβ, and C′ nuclei of free ubiquitin. (TIF) [file pcbi.1002035.s002.tif]

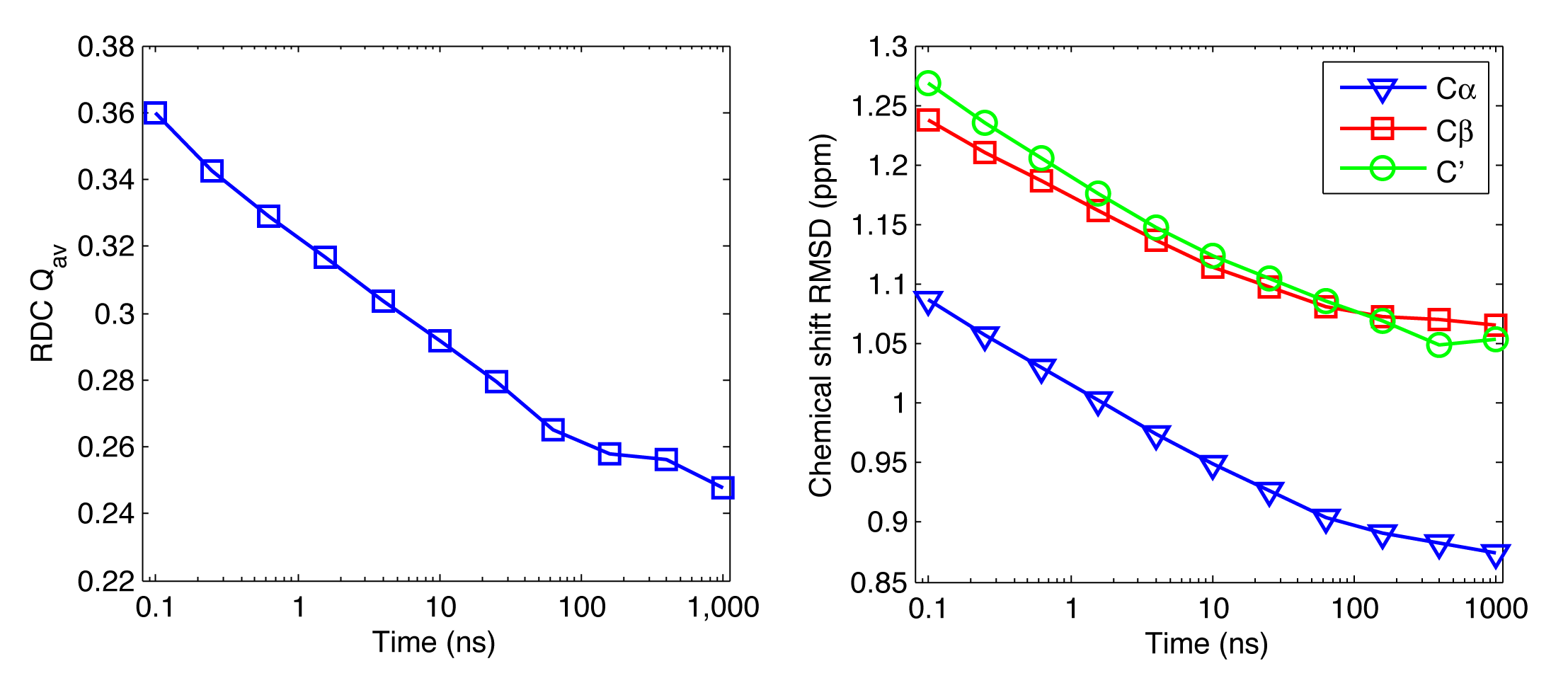

Supplement: Figure S3 — Dependence of NH RDC Qav values (left panel) and chemical shift RMSDs (right panel) as a function of time window size from 0.1 ns to 1 µs for free ubiquitin. (TIF) [file pcbi.1002035.s003.tif]

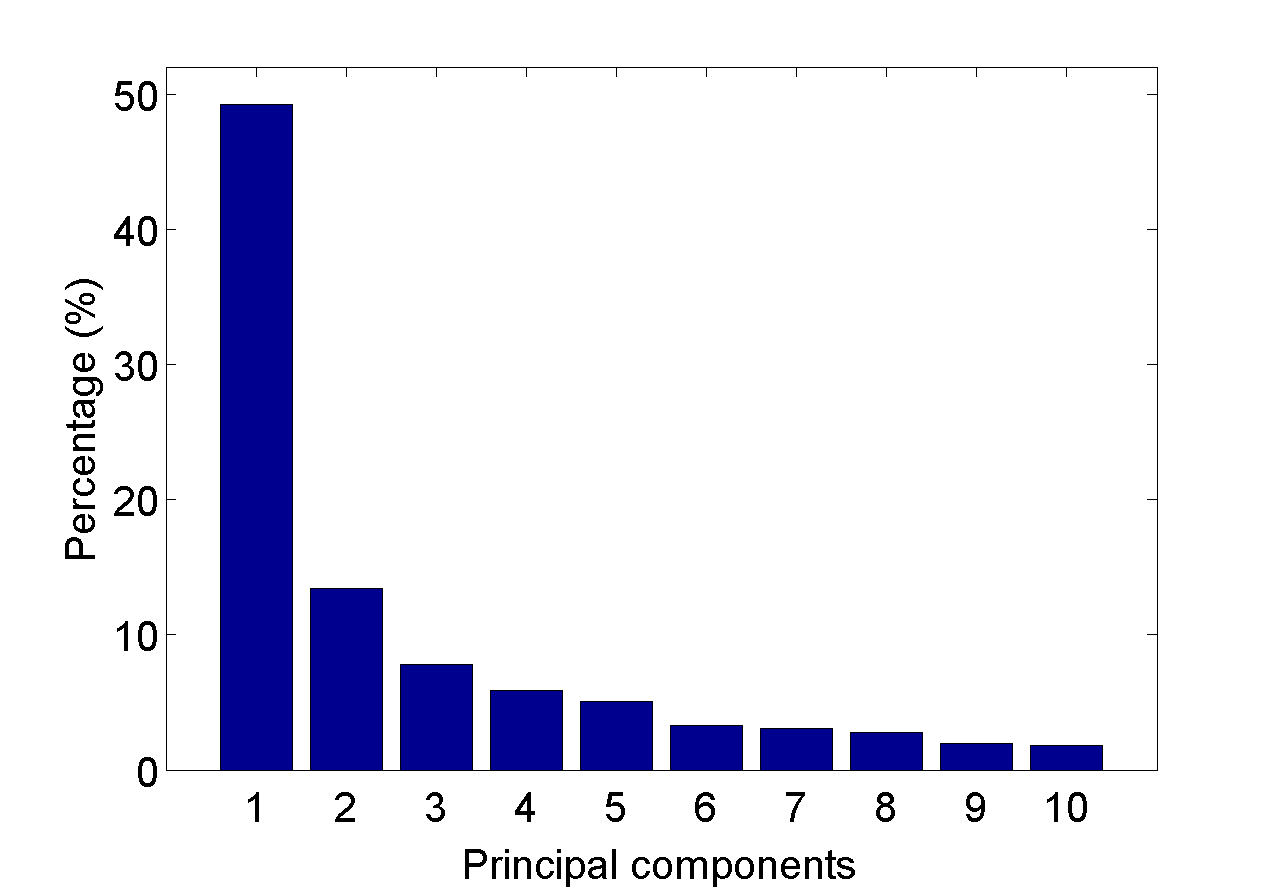

Supplement: Figure S4 — PCA eigenvalues, expressed as percentages (i.e. the sum of all eigenvalues is 100%) of the ten largest principal components determined from the 19 X-ray crystal structures. (TIF) [file pcbi.1002035.s004.tif]

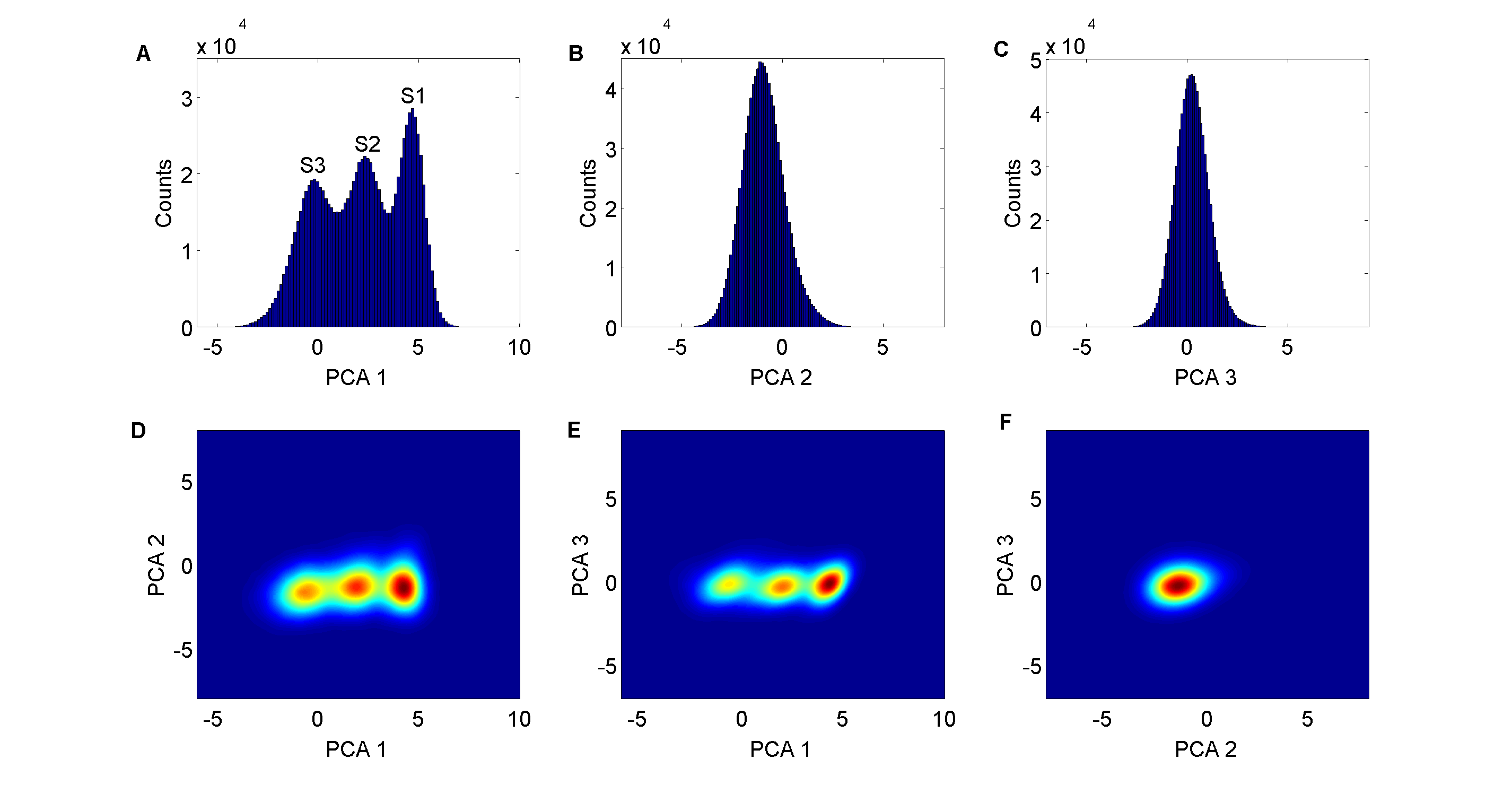

Supplement: Figure S5 — One-dimensional (A–C) and two dimensional (D–F) PCA projections of the 1 µs MD ensemble along the largest principal mode. (TIF) [file pcbi.1002035.s005.tif]

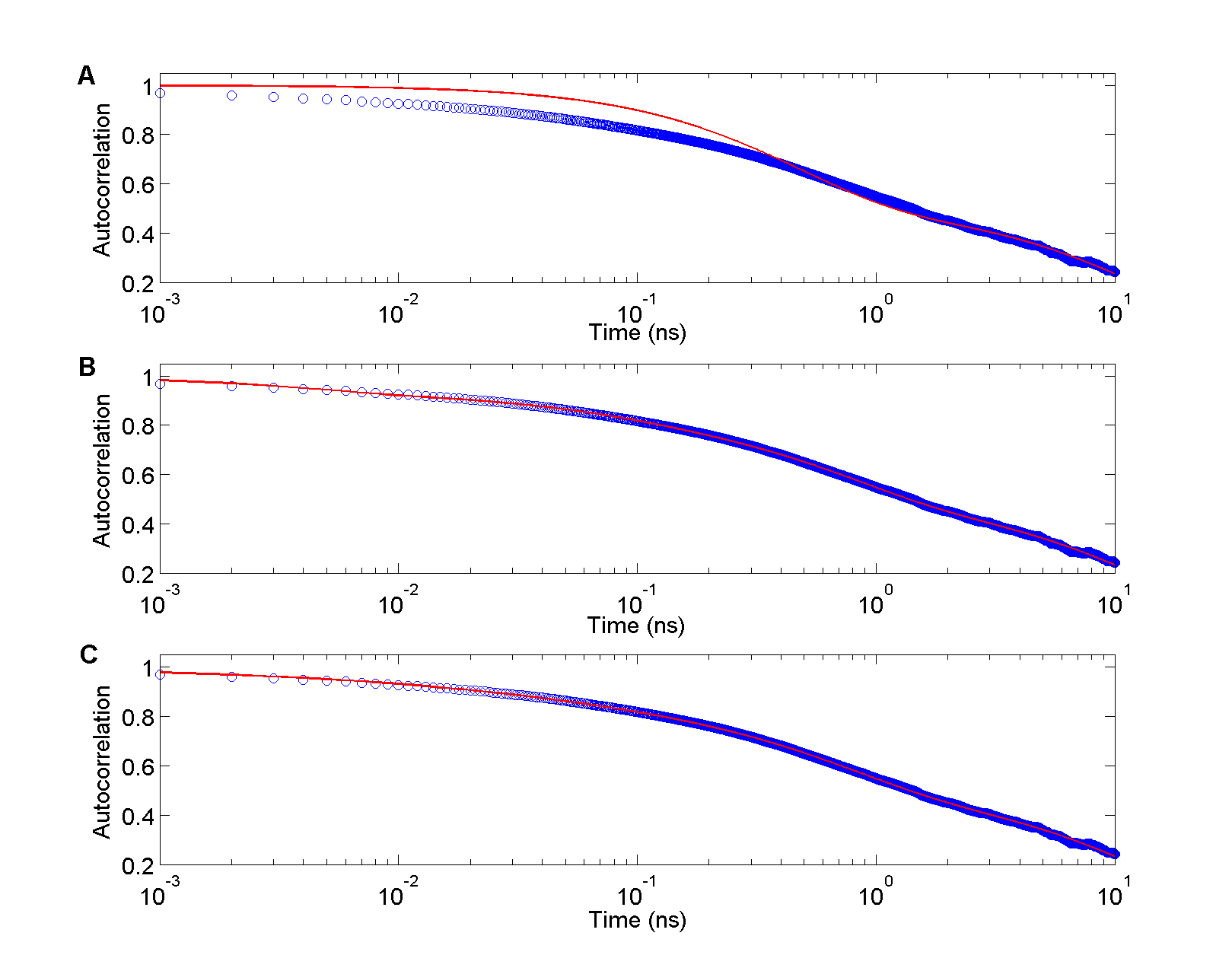

Supplement: Figure S6 — Fitting of the autocorrelation function C(t) (blue open circles) with Eqs. S6 (A), S7 (B), and S8 (C), respectively (red lines). To highlight the quality of fits at short time scales, only the time window <10 ns is displayed. The extracted parameters are (A) Eq. S6: a = 0.49; τfast = 0.4 ns; τslow = 13 ns. (B) Eq. S7: a 1 = 0.07; a 2 = 0.09; a 3 = 0.34; a 4 = 0.5; τ 1 = 0.004 ns; τ 2 = 0.08 ns; τ 3 = 0.7 ns; τ 4 = 13 ns. (C) Eq. S8: a 1 = 0.17; a 2 = 0.33; a 3 = 0.5; τ 1 = 0.04 ns; τ 2 = 0.7 ns; τ 3 = 13 ns; β = 0.54. (TIF) [file pcbi.1002035.s006.tif]

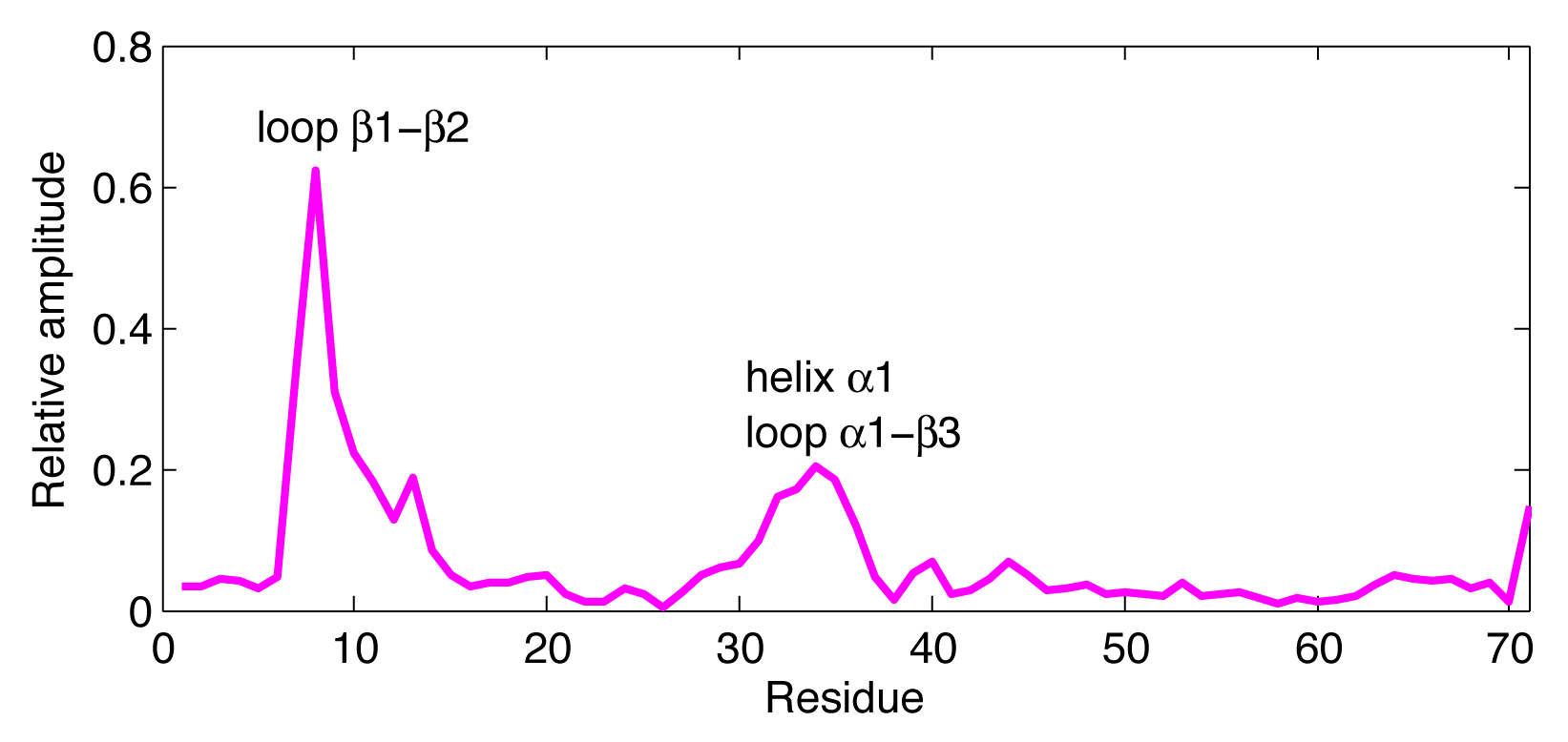

Supplement: Figure S7 — Relative amplitude of Cα positional changes in X-ray structures in the 1st principal component. (TIF) [file pcbi.1002035.s007.tif]

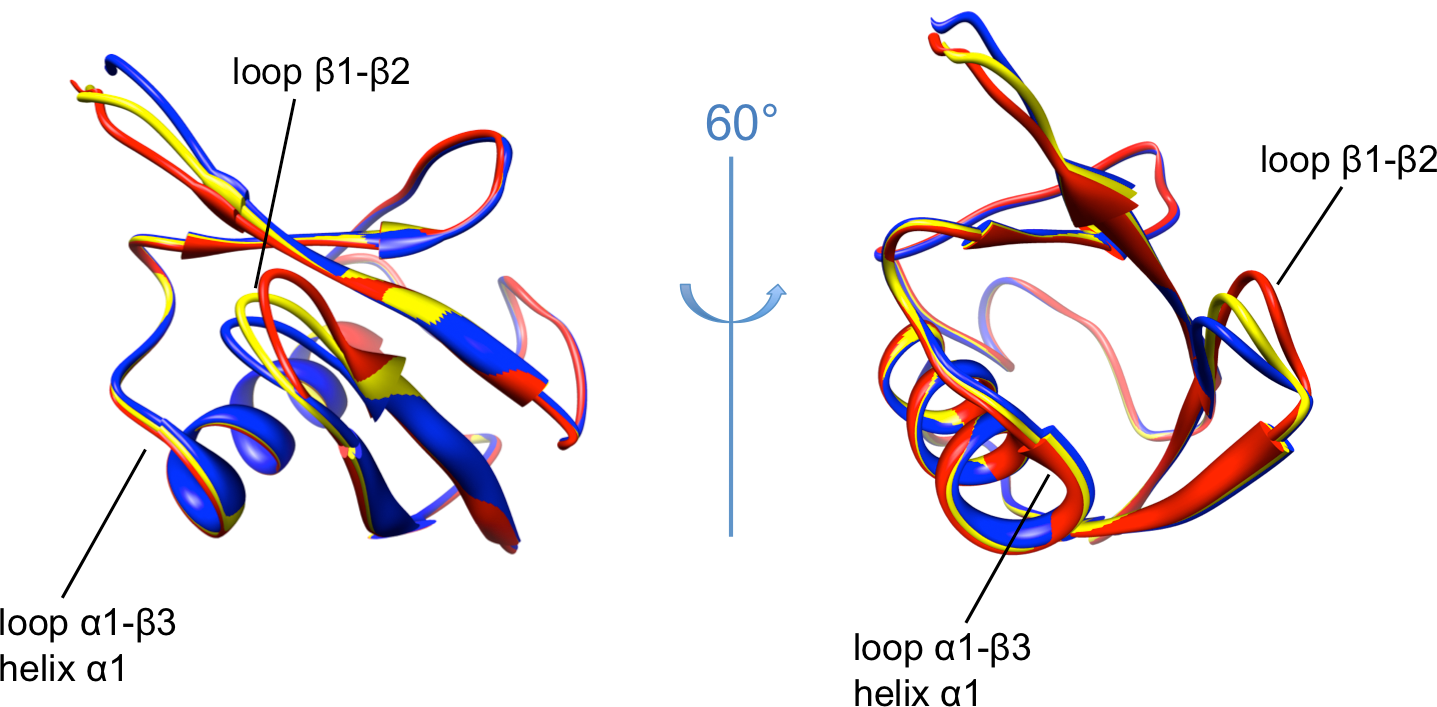

Supplement: Figure S8 — Superposition of ribbon representations of the average structures of substates S1 (blue), S2 (yellow), and S3 (red). The conformers within 0.3 Å of the individual maxima of the three substates depicted in Figure S5A were clustered and used for the determination of average structures. (TIF) [file pcbi.1002035.s008.tif]

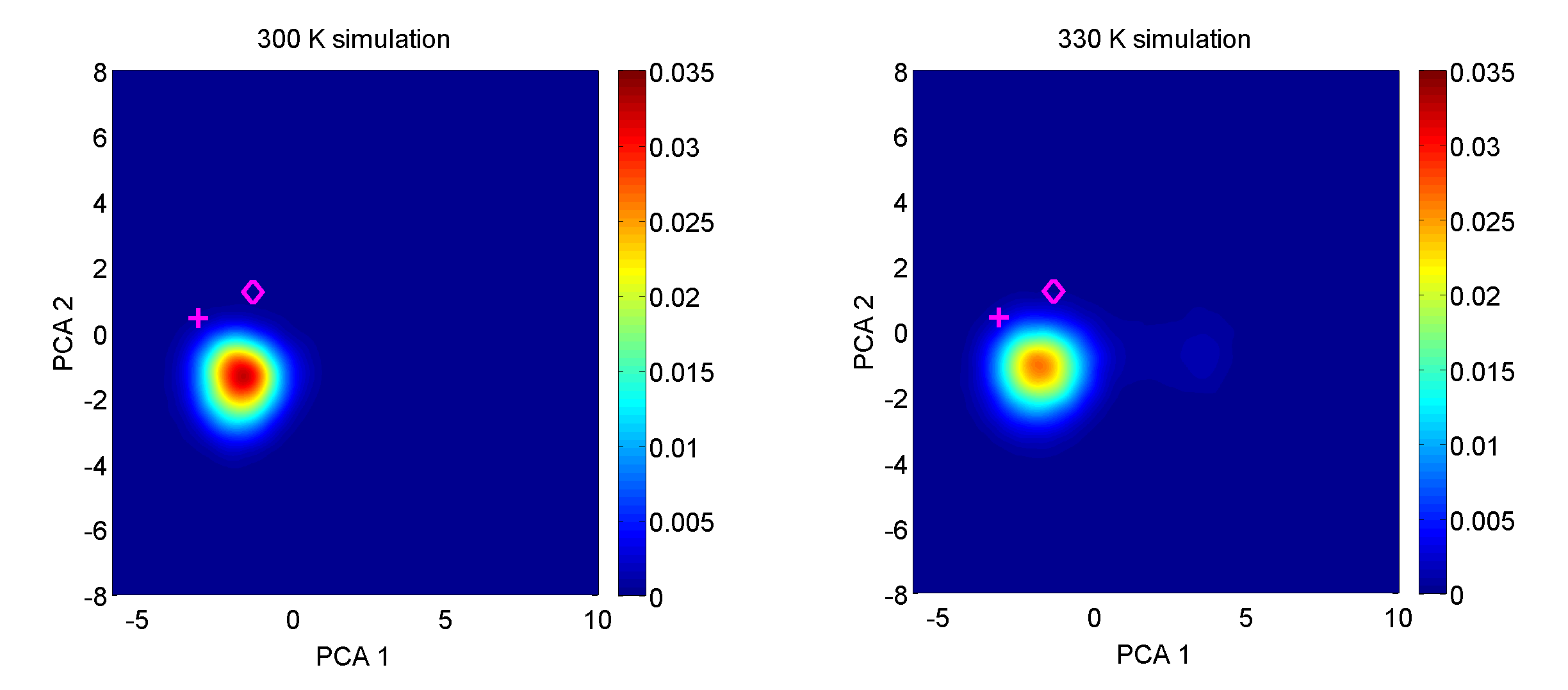

Supplement: Figure S9 — Population distribution of ubiquitin bound to Hrs-UIM sampled at a temperature of 300 K (left panel) and 330 K (right panel). The corresponding crystal bound ubiquitin conformations are indicated by the magenta diamond (for chain A, PDB code: 2D3G) and the magenta ‘+’ symbol (for chain B, PDB code: 2D3G). (TIF) [file pcbi.1002035.s009.tif]

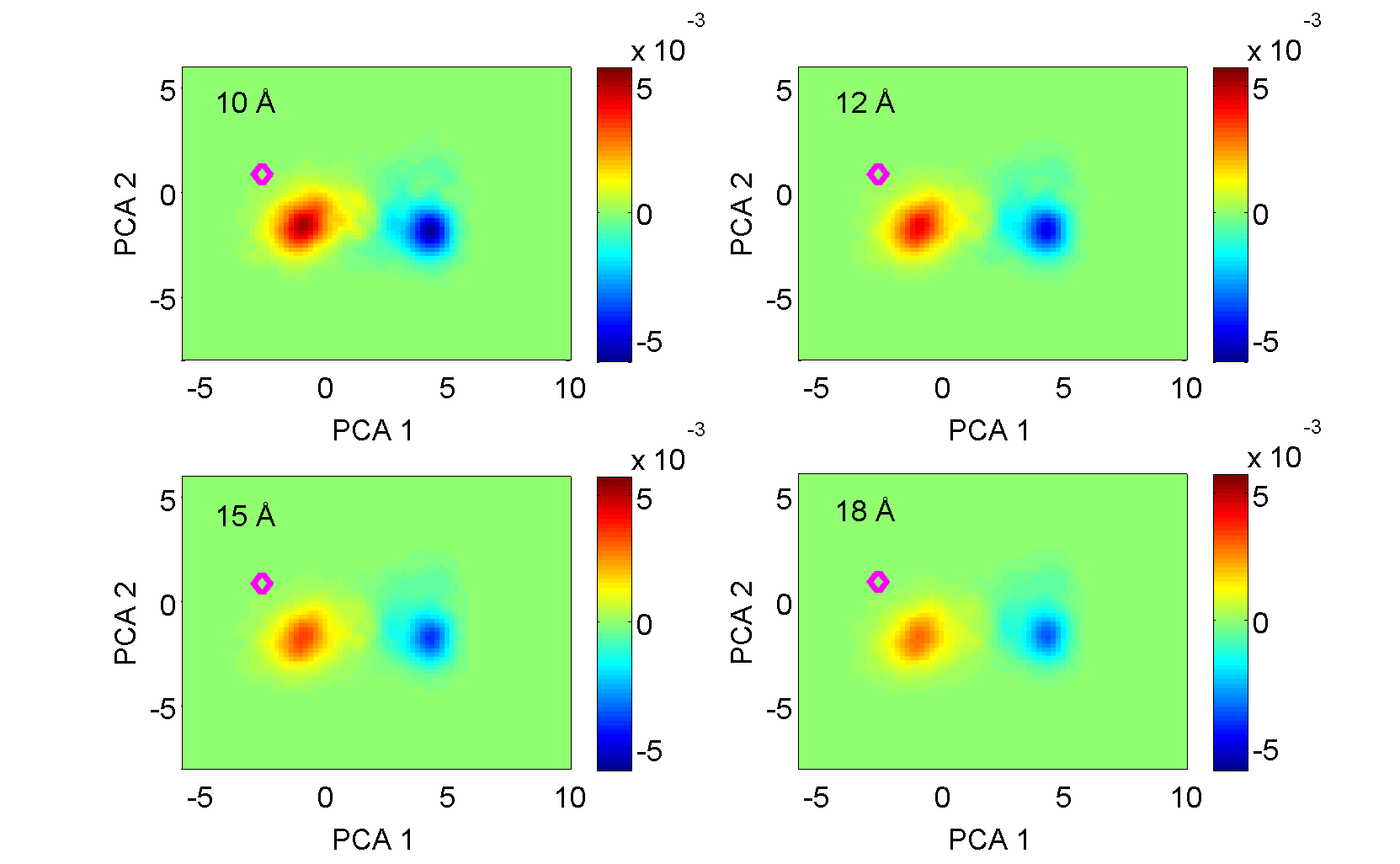

Supplement: Figure S10 — Change of ubiquitin populations upon the approach of human GGA3 GAT domain. Difference maps of populations were calculated at 4 distances from 10–18 Å. The crystal conformation (PDB code: 1YD8, chain U) is indicated by the magenta diamond. (TIF) [file pcbi.1002035.s010.tif]

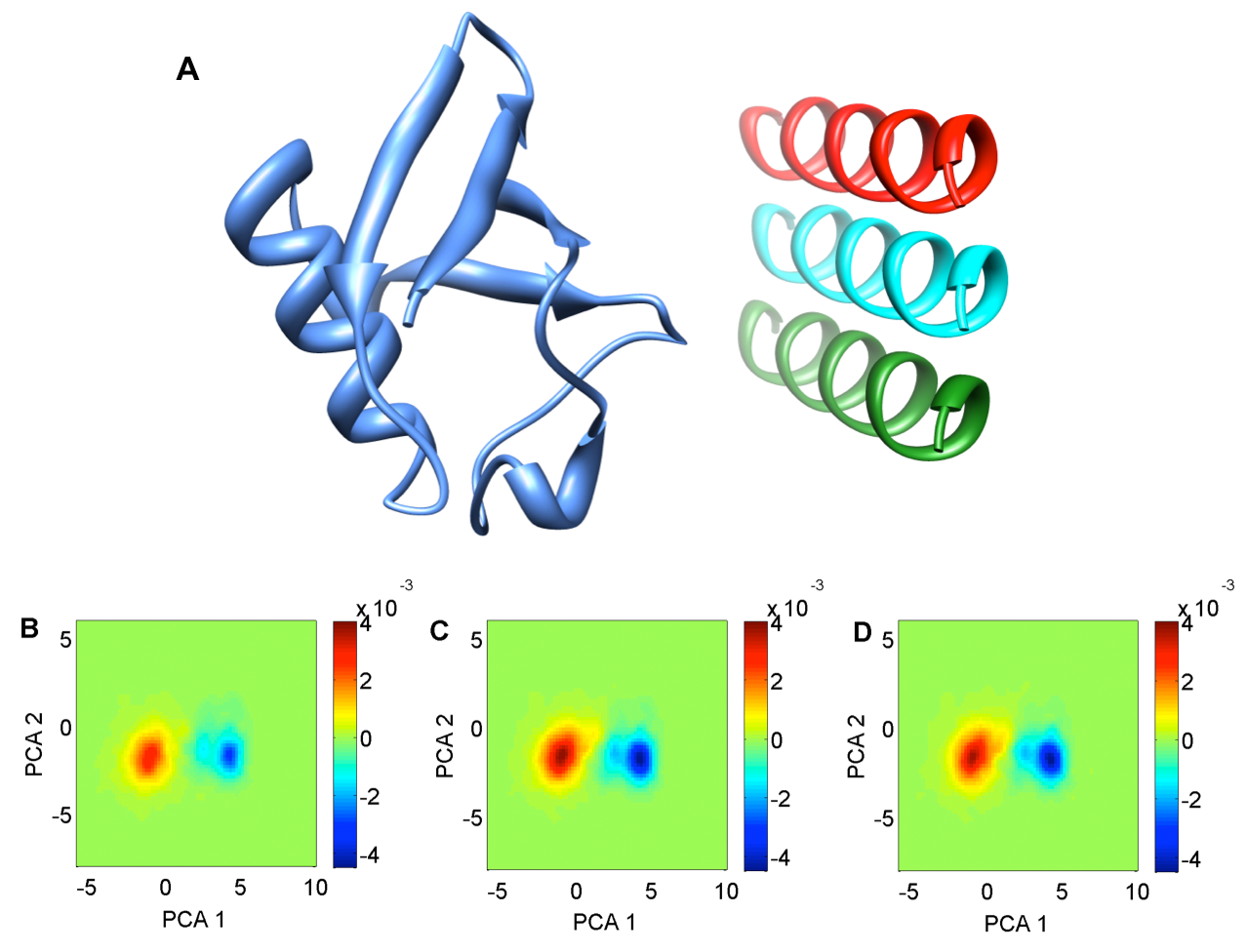

Supplement: Figure S11 — Population shift of ubiquitin upon the approach of Hrs-UIM from different directions. The ligand was placed in three different directions with respect to ubiquitin (panel A), as colored in red, cyan, and green. The corresponding population difference maps are shown in panels B, C, and D, respectively. (TIF) [file pcbi.1002035.s011.tif]

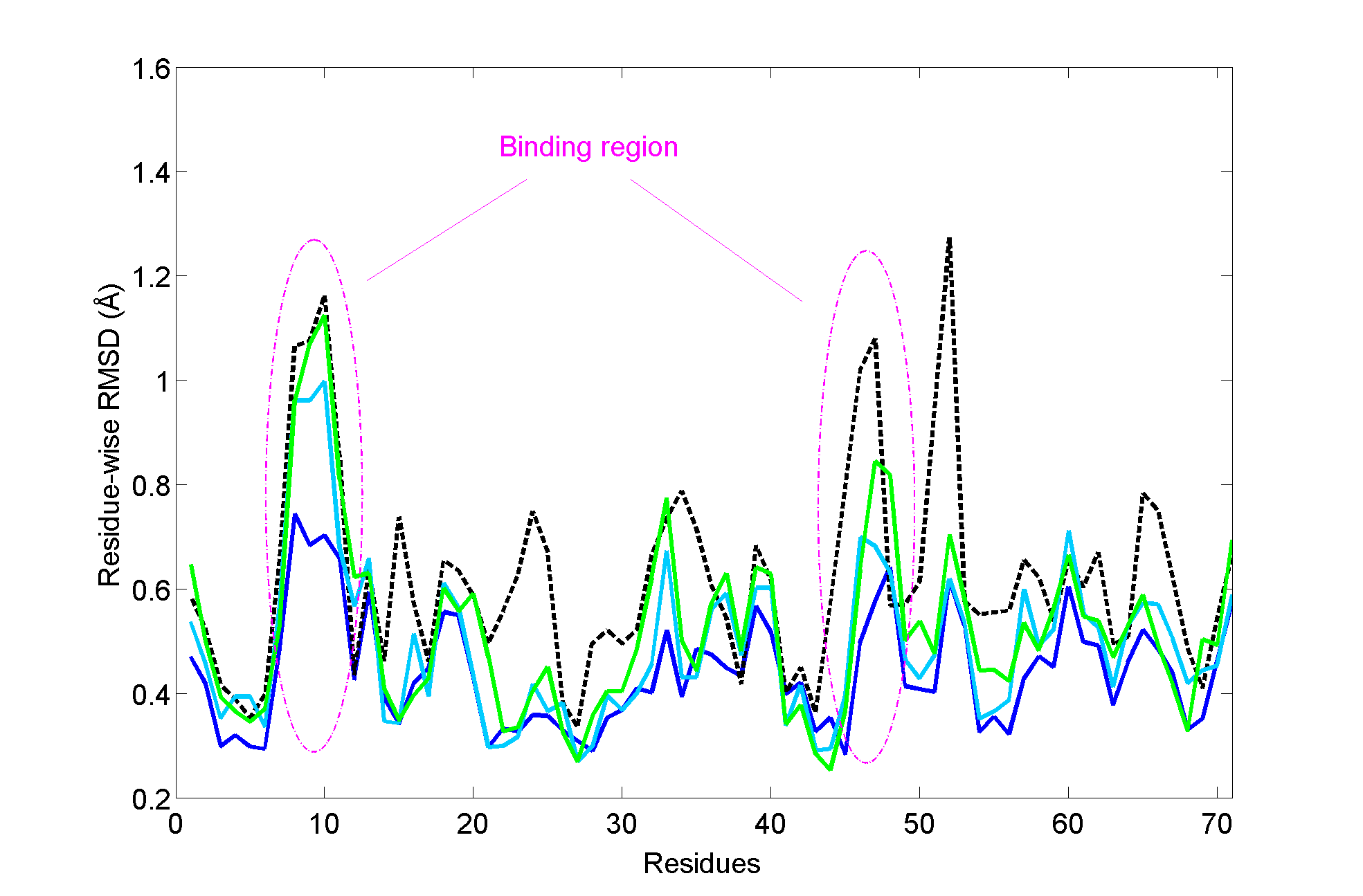

Supplement: Figure S12 — Residue-wise RMSD between X-ray structures with EROS ensemble (black dashed line) and MD ensembles represented by 1 million (blue solid line), 1000 (light blue), and 100 (green) structures. The three MD ensembles were generated by selecting conformations every 1 ps, 1 ns, and 10 ns, respectively. The binding regions are indicated by magenta dash-dot lines. (TIF) [file pcbi.1002035.s012.tif]
