# Supplementary material for: In Silico Elucidation of the Recognition Dynamics of Ubiquitin
Source: PLoS Comput Biol. 2011 Apr 21;7(4):e1002035. doi: 10.1371/journal.pcbi.1002035 (PMC3080845; doi:10.1371/journal.pcbi.1002035)
Supplement: Text S1 — Supplementary methods and supplementary results. (DOC) [file pcbi.1002035.s013.doc]

**Supporting Information**

**Definitions of RMSF and RMSD parameters for structural comparison in Figure 1 of main text**

The root-mean-square-fluctuation (RMSF) (Figure 1A) of an ensemble, after superposition of each conformer onto the reference structure 1UBQ, is calculated as

$RMSF_{k}=\sqrt{\frac{1}{M}\sum_{m=1}^{M} d_{m,k}^{2}}$ (S1)

where *d_m,k_* is the distance between the Cα atom of the *k*th residue of the *m*th structure of the ensemble consisting of M structures and that of the average structure of the ensemble.

For pair-wise structural comparison, the overall Cα root-mean-square-deviation (RMSD) between two structures from the X-ray ensemble and the MD (or EROS) ensemble is calculated as

$RMSD=\sqrt{\frac{1}{K}\sum_{k=1}^{K} d_{k}^{2}}$ (S2)

where *K* = 71 is the number of residues (without the flexible C-terminal tail), *d_k_* is the distance between the Cα atoms of the *k*th residue of the two structures compared to each other. The structures in the MD (or EROS) ensemble, which shows the lowest overall RMSD with individual X-ray crystal structures, are selected (Figure 1C) and subjected to the calculation of a residue-wise RMSD (Figure 1B) (see also Method section of main text),

$RMSD_{k}=\sqrt{\frac{1}{19}\sum_{i=1}^{19} d_{i,k}^{2}}$ (S3)

where *d_i,k_* is the distance between the Cα atom of the *k*th residue of the *i*th X-ray structure and that of the corresponding closest conformer in the MD or EROS ensembles, respectively.

**Direct validation of 1 μs trajectory against experimental NMR data**

The residual dipolar couplings (RDCs) were back-calculated from the 1 μs MD trajectory using the method described previously [1] and compared with experimental values [2] (Figure S1). The average RDC-derived Q-value, *Q_av_*, is defined in standard fashion as:

$Q_{av}=\frac{1}{N}\sum_{n=1}^{N} \frac{\sqrt{mean[(RDC_{calc}-RDC_{\exp})^{2}]}}{\sqrt{mean[RDC_{\exp}^{2}]}}$ (S4)

where *mean* denotes the average over the dipolar vectors and *N* is the number of different alignment media. Conformationally averaged chemical shifts of ubiquitin were predicted from the 1 μs trajectory using SHIFTS [3] and compared with the experimental data (Figure S2). The dependence of RDC *Q_av_* values and chemical shift RMSD values on the length of the time-averaging window (Figure S3) was calculated as described previously [1,4].

**Correlation time of the internal dynamics along the first principal axis**

The autocorrelation function of the largest principal component is calculated as,

(S5)

where *x(t)* is the projection of snapshot at time t on the first principal component and *μ* is the mean value. The angular brackets denote ensemble averaging. is the variance of the distribution. The autocorrelation function is then fitted using three different analytical forms (Eqs. S6-S8),

(S6)

where *τ_fast_* and *τ_slow_* are two effective correlation times and *a* is the weight of fast motions (0 < *a* < 1).

(S7)

where *τ_n_* represents effective correlation times on four different time regimes (*τ*_1_ < *τ*_2_ < *τ*_3_ <*τ*_4_). *a_n_* are the corresponding weights, satisfying and .

(S8)

where a stretched exponential function (the first term) is included to describe the rapid decay with exponent *β* (0< *β* <1) [5]. The coefficients *a_n_* are the corresponding weights.

In all models, a slowly decaying exponential with a correlation time of 13 ns yields the largest contribution (50%). In the more sophisticated models of Eqs. S7 & S8, an additional exponential with an effective correlation time of 0.7 ns is identified. The decay of the autocorrelation function on fast time scales (< 100 ps) requires for its characterization at least two additional discrete correlation times (4 ps and 80 ps), or alternatively, a stretched exponential function with an exponent of *β* = 0.54 (see Figure S6).

**Principal mode motion of ubiquitin**

The relative amplitudes **a** of positional changes of individual Cα atoms along the largest principal mode (Figure S7) are obtained from the associated eigenvector of the Cartesian 3n×3n covariance matrix as:

(S9)

where *n* is the total number of residues analyzed.

This principal mode motion of free ubiquitin reflects a collective pincer-like motion as originally proposed by Lange et al. [6]. To visually illustrate the principal mode motion of free ubiquitin in the MD simulation, the average structures of the three substates S1, S2 and S3 are superimposed for comparison in Figure S8.

**Investigation of the accessible conformational space of ubiquitin bound with Hrs-UIM.**

Although the bound forms of ubiquitin are represented as static structures in crystallographic studies, it is expected that under ambient conditions they undergo dynamics sampling a range of conformations. To verify the convergence and stability of sampling by the MD trajectory at 300 K (see Figure 4 in main text), a second MD simulation of ubiquitin:Hrs-UIM was performed for 300 ns at an elevated temperature of 330 K.

The conformational space sampled at the two different temperatures is very similar (Figure S9). Both distributions show significant overlap with the S3 state of free ubiquitin (which is the state that shows an increase in population upon the approach of UIM (Figure 5 in the main text)).

**Population shift of ubiquitin upon the approach of Hrs-UIM from different directions.**

Being a relatively small and structurally well-defined ligand, the optimal pathway for Hrs-UIM to approach the binding interface of ubiquitin is rather straightforward. Therefore, in the present study (main text), the protein and ligand molecules were placed in a relatively favorable orientation, and the analysis of population shift mainly focused on its distance-dependence.

In order to test the robustness of the observed results, the effect of different orientations is also investigated here. For a single distance (12 Å), the ligand molecule was positioned in three different directions (Figure S11A), with *∠*P_1_OP_2_ = 15° and *∠*P_1_OP_3_ = 30°, where O is the centre of mass (COM) of ubiquitin, and P_1_/P_2_/P_3_ is the COM of Hrs-UIM in three positions (red/cyan/green). The corresponding population difference maps were subsequently calculated (Figure S11B-C) for the three directions. Although there is some quantitative difference of the three population maps, the overall trend of population shift (from substates S1 and S2 to substate S3) is preserved for all three directions.

**References**

1. Showalter SA, Brüschweiler R (2007) Quantitative molecular ensemble interpretation of NMR dipolar couplings without restraints. J Am Chem Soc 129: 4158-4159.

2. Lakomek NA, Walter KF, Fares C, Lange OF, de Groot BL et al. (2008) Self-consistent residual dipolar couplings based model-free analysis for the robust determination of nanosecond to microsecond protein dynamics. J Biomol NMR 41: 139-155.

3. Xu XP, Case DA (2001) Automated prediction of 15N, 13Calpha, 13Cbeta, and 13C' Chemical shifts in proteins using a density functional database. J Biomol NMR 21: 321-333.

4. Li D-W, Brüschweiler R (2010) Certification of molecular dynamics trajectories with NMR chemical shifts. J Phys Chem Lett 1: 246-248.

5. Calandrini V, Abergel D, Kneller GR (2010) Fractional protein dynamics seen by nuclear magnetic resonance spectroscopy: Relating molecular dynamics simulation and experiment. J Chem Phys 133: 145101.

6. Lange OF, Lakomek NA, Fares C, Schroder GF, Walter KF et al. (2008) Recognition dynamics up to microseconds revealed from an RDC-derived ubiquitin ensemble in solution. Science 320: 1471-1475.
